# Omega-3 Polyunsaturated Fatty Acid Formulations in Cardiovascular Prevention: Balancing Clinical Efficacy, Safety, and Environmental Sustainability

**DOI:** 10.3390/nu18152538

**Published:** 2026-08-04

**Authors:** Filippo Egalini, Enrico Riccio, Marco Grasso, Anna Nelva, Vincenzo De Geronimo

**Affiliations:** 1 Lipidology and Metabolism Commission Associazione Medici Endocrinologi, 29122 Piacenza, Italy; 2 Division of Endocrinology, Department of Multidimensional Medicine, AUSL Toscana Centro, 50122 Firenze, Italy; 3 Endocrine, Metabolism and Nutrition Disease Unit, Santa Maria Di Ca’ Foncello Hospital, 31100 Treviso, Italy; 4 Lipidology and Metabolism Commission Associazione Medici Endocrinologi, 13900 Biella, Italy; 5 Centro Clinico Diagnostico GB Morgagni Srl, 95125 Catania, Italy

**Keywords:** eicosapentaenoic acid, docosahexaenoic acid, cardiovascular risk, Omega-3 index, microalgae, environmental sustainability

## Abstract

Omega-3 polyunsaturated fatty acids (ω-3 PUFAs), specifically eicosapentaenoic acid (EPA) and docosahexaenoic acid (DHA), are critical modulators of cardiovascular physiology, acting through anti-inflammatory signaling, membrane remodeling, and transcriptional regulation. Despite strong biological plausibility, clinical evidence remains heterogeneous. This narrative review evaluates the complexities of ω-3 supplementation, moving beyond standardized dosing to discuss the emerging role of biomarker-based assessments. While traditional low-dose mixed formulations often failed to show significant benefits, high-dose purified EPA (icosapent ethyl, 4 g/day) has demonstrated a substantial reduction in residual cardiovascular risk in specific populations. However, this clinical success is accompanied by a modest but consistent increase in atrial fibrillation risk at pharmacological doses, suggesting a need for better risk stratification. We highlight that therapeutic efficacy is closely tied to bioavailability—driven by chemical form and dietary context. In this setting, objective metrics such as the ω-3 Index or EPA/arachidonic acid (AA) ratio are discussed as validated biomarkers of ω-3 status that, although not yet incorporated into clinical guidelines as routine treatment targets, may help account for inter-individual variability. Furthermore, we address the environmental burden of marine-derived oils, considering microalgae-based alternatives as sustainable solutions. Ultimately, this review advocates for a more nuanced approach that integrates clinical evidence, the potential of biomarker monitoring, and global environmental responsibility.

## 1. Background

In recent years, Omega-3 (ω-3) polyunsaturated fatty acids (PUFAs) have gained considerable attention due to their involvement in promoting health and reducing the risk of several diseases. This group includes α-linolenic acid (ALA; C18:3 ω-3), stearidonic acid (SDA; C18:4 ω-3), eicosapentaenoic acid (EPA; C20:5 ω-3), docosapentaenoic acid (DPA; C22:5 ω-3), and docosahexaenoic acid (DHA; C22:6 ω-3). These fatty acids are mainly derived from plant sources or produced through plant modification processes, as well as from marine organisms, algae, and single-cell sources [1].

Among these, EPA and DHA are the most extensively investigated ω-3 PUFAs. In addition to maintaining cellular membrane fluidity, they serve as precursors for specialized pro-resolving mediators (SPMs) that drive anti-inflammatory processes, thereby contributing to cardiovascular and neurological health [2].

The intake of long-chain ω-3 PUFAs (ω-3 LCPUFAs) largely depends on dietary patterns, resulting in significant variability across populations and geographical regions [3].

Linoleic acid (LA; C18:2 ω-6), the predominant ω-6 PUFA, is mainly present in vegetable oils, nuts, and oil-rich seeds. Within the body, LA can be metabolized into γ-linolenic acid (GLA; C18:3 ω-6), which is further converted into dihomo-γ-linolenic acid (DGLA; C20:3 ω-6) and subsequently into arachidonic acid (AA; C20:4 ω-6). In addition to endogenous synthesis, AA is also obtained from animal-derived foods such as meat, eggs, and fish [3]. While AA predominantly drives the synthesis of pro-inflammatory eicosanoids, ω-3 PUFAs exert opposing, anti-inflammatory effects by competing with AA for metabolism, thereby modulating the overall inflammatory response.

ω-3 and ω-6 PUFAs cannot be interconverted and must be obtained through the diet; the balance between these two families has become a key focus in reducing inflammation and lowering the risk of metabolic diseases [4]. ω-6 and ω-3 PUFAs share the same desaturation and elongation enzymes during the endogenous conversion of their essential precursors (linoleic acid and α-linolenic acid), resulting in metabolic competition. However, this competition primarily affects the limited conversion of α-linolenic acid to EPA and DHA and is largely bypassed when preformed marine-derived EPA and DHA are consumed. The clinical significance of the ω-6/ω-3 ratio remains controversial, and many experts consider absolute EPA and DHA status, such as the Omega-3 Index, to be a more informative biomarker [5,6]. Within this debate, however, the EPA/AA ratio retains clinical utility as a more refined, mechanistically grounded variant of the ω-6/ω-3 concept: unlike the generic ratio (which aggregates multiple, functionally heterogeneous ω-6 and ω-3 species), EPA/AA isolates the two fatty acids that directly compete for the same desaturase and cyclooxygenase/lipoxygenase enzymes, making it a more specific marker of the eicosanoid-mediated inflammatory balance [7,8]. Higher dietary linoleic acid intake does not necessarily increase tissue arachidonic acid levels or systemic inflammation in humans [9].

Historically, human diets maintained a relatively balanced ω-6/ω-3 ratio. However, the rapid dietary changes that have occurred over the past 100–150 years represent a relatively recent phenomenon in human evolution. Recommended intake levels correspond to 0.5% of total energy for ALA and 4% for LA. However, beneficial effects of ALA are generally observed at slightly higher intake levels (0.6–1% of total energy) [10].

ω-3 PUFAs are mainly found in aquatic organisms. They originate from the liver of lean fish such as cod and halibut, from the tissues of fatty fish including mackerel, menhaden, and salmon, and from the adipose tissues of marine mammals such as seals and whales [11]. Marine sources are particularly rich in EPA and DHA, while DPA is generally present in smaller amounts in most fish oils [11].

Plant-based foods represent the primary source of ALA, which is concentrated in certain seeds, nuts, and vegetable oils. Flaxseed, chia seeds, walnuts, and echium oil are notable sources of ALA, whereas safflower, sunflower, corn, and soybean oils are rich in linoleic acid (18:2 ω-6) [12].

Marine oils are also important sources of fat-soluble vitamins. The livers of species such as cod, haddock, halibut, shark, whale, and tuna are commonly used for the production of vitamin A and D supplements. Cod liver oil contains approximately 1000 IU/g of vitamin A and 100 IU/g of vitamin D [1].

### Food Sources, Intake Adequacy and Recommendations

Although ALA is widely distributed in plant foods, flaxseed oil being especially rich (typically ranging from 45.0 to 55.0 g/100 g depending on the cultivar and environmental growth conditions) [13], preformed long-chain ω-3 PUFAs are concentrated in marine foods, which remain the principal dietary source of EPA and DHA. Among fish, cod, halibut, and skipjack tuna can reach high DHA contents (up to 30% of total fatty acids), while cod, flounder, and haddock are characterized by high EPA contents (15–19% of total fatty acids) [12]. On a per-portion basis, oily and processed marine products provide substantial amounts: salted mackerel, for example, has been reported to contain 4.57 g of EPA + DHA per 100 g of cooked sample [1]. Cod liver oil is a further notable source, also rich in fat-soluble vitamins (approximately 1000 IU/g vitamin A and 100 IU/g vitamin D) [1]. The per-food and per-portion contributions are summarized in Table 1.

Actual dietary intake of EPA and DHA in the general population is frequently below recommended levels. In the National Health and Nutrition Examination Survey (NHANES 2003–2008), mean intake from food alone in U.S. adults was 23 ± 1 mg/day of EPA and 63 ± 2 mg/day of DHA, rising to 41 ± 4 and 72 ± 4 mg/day, respectively, when supplement use was included; mean ALA intake from food in adults aged ≥19 years was 1.5 ± 0.01 g/day [15]. This shortfall is global in scale: a systematic analysis spanning 187 countries documented seafood-derived EPA + DHA intake below recommended levels in the large majority of the world’s adult population [16]. Baseline EPA/DHA status also influences responsiveness to supplementation in randomized trials and the generalizability of their findings across populations with different dietary patterns (see Section 3 and Section 6).

Quantitative recommendations for EPA and DHA intake vary substantially across scientific and health organizations. A recent systematic review of 42 technical and scientific documents (TSDs) underpinning national and international dietary guidelines found that 71% contained quantitative recommendations, with marked heterogeneity in values, units, and target age groups, and a median recommendation of 313 mg/day among documents framed around a health benefit [17]. For the general adult population, the most frequently reported figure is 250 mg/day of EPA + DHA, consistent with the EFSA adequate intake [18]. Several bodies propose higher, cardiovascular-oriented thresholds: the International Society for the Study of Fatty Acids and Lipids (ISSFAL) recommends a minimum of 500 mg/day of EPA + DHA for cardiovascular health [19], while France (Agence nationale de sécurité sanitaire de l’alimentation, de l’environnement et du travail or ANSES, 500 mg/day) [20]. The United Kingdom (Scientific Advisory Committee on Nutrition or SACN, ≥450 mg/day, equivalent to about two portions of fish per week including one oily) [21] set values above the EFSA adequate intake. During pregnancy and lactation, the common recommendation is 250 mg/day of EPA + DHA plus an additional 100–200 mg/day of DHA [18]. With respect to safety, supplemental intakes of up to 5 g/day EPA + DHA, 1.8 g/day EPA alone, and approximately 1 g/day DHA alone have been judged to raise no safety concerns in the general population [22,23]. These recommendations are collated in Table 2. Overall, the contrast between nutritional reference thresholds (250–500 mg/day) and the pharmacological doses used in clinical trials (1.8–4 g/day; see Table 3) reflects a categorical distinction rather than a single dose–response continuum. While nutritional guidelines aim to maintain ω-3 PUFA status in healthy individuals, pharmacological interventions target the reduction in residual cardiovascular risk using high-purity EPA.

## 2. Pharmacological and Molecular Mechanisms of ω-3 PUFAs

The effects of marine ω-3 PUFAs on inflammation and immune cell function have been extensively studied, demonstrating a wide range of biological activities. Overall, the evidence suggests that these fatty acids exert anti-inflammatory effects and may also contribute to the resolution phase of inflammation.

Chemotaxis, the process by which leukocytes migrate toward sites of inflammation in response to chemical signals, is one of the key mechanisms affected. Chemoattractants such as leukotriene B4 (LTB4), derived from arachidonic acid, play a major role in this process. Studies in healthy individuals supplemented with fish oil have shown a reduction in the chemotactic activity of neutrophils and monocytes toward various stimuli, including LTB4, bacterial peptides, and human serum [13].

Adhesion molecules, expressed on the surface of endothelial cells and leukocytes, facilitate interactions between these cells and allow leukocytes to migrate from the bloodstream to inflamed tissues. Experimental studies in cell cultures [26] and animal models [27] have demonstrated that exposure to marine ω-3 PUFAs reduces the expression of these molecules on monocytes, macrophages, lymphocytes, and endothelial cells. In some cases, this reduction was associated with decreased adhesion between leukocytes and endothelial cells [27].

In human studies, supplementation with fish oil providing approximately 1.5 g/day of EPA and DHA has been associated with reduced expression of intercellular adhesion molecule-1 (ICAM-1) on stimulated monocytes [28]. Similarly, intake of 1.1 g/day of EPA and DHA has been shown to reduce circulating levels of soluble vascular cell adhesion molecule-1 (VCAM-1) in elderly individuals [29]. In patients with metabolic syndrome, EPA supplementation (1.8 g/day) resulted in decreased levels of both soluble ICAM-1 and VCAM-1 [27].

Eicosanoids, which are lipid mediators derived from 20-carbon PUFAs, play a central role in inflammation. These molecules are primarily synthesized from arachidonic acid, which is released from membrane phospholipids by phospholipase A2 enzymes in response to inflammatory stimuli. Arachidonic acid is then metabolized by cyclooxygenase (COX), lipoxygenase (LOX), and cytochrome P450 enzymes to generate prostaglandins, thromboxanes, leukotrienes, and other derivatives. These mediators act through specific receptors, often G protein-coupled receptors, and are key regulators of inflammatory processes [30].

EPA can also serve as a substrate for these enzymatic pathways. However, the eicosanoids derived from EPA are generally less biologically active than those produced from arachidonic acid [31]. This reduced activity is partly due to a lower affinity of receptors for EPA-derived mediators. For instance, prostaglandin E3 (PGE3) has been shown to exhibit 50–80% lower potency compared to PGE2 at various receptor subtypes [32]. As a result, increased EPA availability leads to reduced production of potent arachidonic acid-derived mediators and increased production of less active compounds.

Several studies have demonstrated that fish oil supplementation reduces the production of pro-inflammatory eicosanoids such as PGE2 and leukotrienes [33]. Additionally, ω-3 PUFAs have been shown to inhibit the production of pro-inflammatory cytokines, including TNF-α, IL-1β, and IL-6, in both in vitro and human studies [34].

PUFAs are essential structural components of membrane phospholipids. In inflammatory cells, membranes typically contain high levels of arachidonic acid and relatively low amounts of EPA and DHA [33]. However, dietary supplementation with fish oil leads to increased incorporation of EPA and DHA into cell membranes, accompanied by a reduction in arachidonic acid content. The incorporation of ω-3 PUFAs into cell membranes is dose-dependent [35] and strictly dependent on the turnover kinetics of each cell type. In erythrocytes (mean lifespan ~120 days), the time required to reach a new steady state after a change in EPA and DHA intake is on the order of 90–196 days for the ω-3 [36]. In leukocytes (hours to days), changes may become apparent more rapidly, whereas in long-lived cells such as neurons, the process is considerably slower. The timeframe of incorporation therefore cannot be generalized to “days” but must be referred to the specific cell type and its lifespan. These changes alter the availability of substrates for the synthesis of bioactive lipid mediators, including eicosanoids, resolvins, protectins, and endocannabinoids.

Another mechanism involves lipid rafts, specialized membrane microdomains involved in signal transduction. In T lymphocytes, these structures are essential for proper cellular activation. Studies have shown that ω-3 PUFAs modify the composition and function of lipid rafts, impairing intracellular signalling and reducing T-cell responsiveness [37].

At the transcriptional level, ω-3 PUFAs influence nuclear factor kappa B (NF-κB), a key regulator of inflammatory gene expression. NF-κB controls the transcription of genes encoding cytokines, adhesion molecules, and enzymes such as COX-2 [38]. Activation of NF-κB involves phosphorylation of its inhibitory subunit (IκB), allowing the transcription factor to translocate to the nucleus [39]. EPA and DHA have been shown to inhibit NF-κB activation by reducing IκB phosphorylation, thereby decreasing inflammatory gene expression [39,40]. In contrast, saturated fatty acids enhance NF-κB activation [41].

G protein-coupled receptors, particularly GPR120, also play a role in mediating the anti-inflammatory effects of ω-3 PUFAs. Activation of GPR120 by EPA and DHA inhibits macrophage responses to inflammatory stimuli, reducing cytokine production and maintaining cytosolic IκB levels [29]. Experimental evidence suggests that the inhibitory effects of DHA on NF-κB activation are mediated through GPR120 [42].

ω-3 PUFAs also regulate lipid metabolism through their effects on sterol regulatory element-binding proteins (SREBPs), which control the expression of genes involved in fatty acid and cholesterol synthesis. These fatty acids suppress SREBP-1 expression at both transcriptional and post-transcriptional levels [43]. This involves interference with liver X receptor (LXR) signalling and increased degradation of SREBP-1 mRNA [44,45]. As a result, the expression of lipogenic enzymes is reduced, leading to decreased hepatic triglyceride synthesis [46].

Finally, peroxisome proliferator-activated receptors (PPARs) are key regulators of lipid metabolism and act as nuclear receptors that control gene expression [47]. ω-3 PUFAs serve as ligands for PPARs, promoting fatty acid oxidation and improving lipid homeostasis [48,49]. Activation of PPARs also enhances the expression of lipoprotein lipase (LPL), facilitating the clearance of triglyceride-rich lipoproteins from circulation [50].

**Figure 1:**
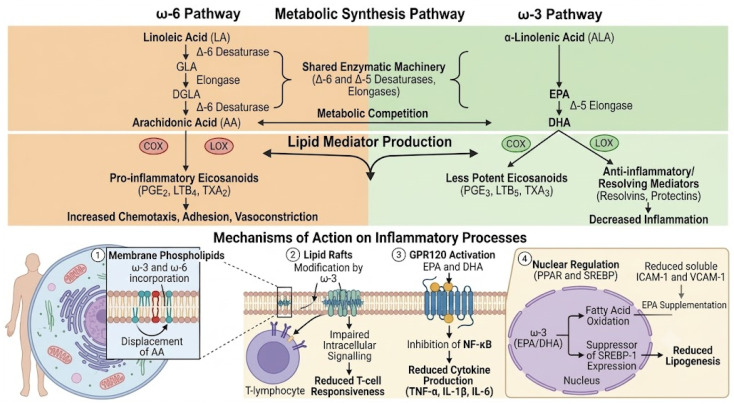
Metabolic pathways of ω-3 and ω-6 PUFAs and their primary molecular mechanisms of action on inflammatory and metabolic processes. The upper panel highlights the enzymatic competition between the two families. The middle panel illustrates the divergence in lipid mediator production (pro-inflammatory vs. anti-inflammatory/resolving). The lower panel summarizes cellular mechanisms, including membrane phospholipid remodeling (1), lipid raft disruption (2), GPR120-mediated NF-kB inhibition (3), and nuclear regulation via PPAR and SREBP-1 (4). Symbols and Legend: Black arrows denote metabolic process flow and downstream biological cascades, while dashed lines indicate structural zoom-in views of the cell membrane.

Fatty acids produced during lipogenesis can undergo desaturation processes; these reactions occur in the so-called microsomal fraction and are carried out by specific oxygenases. In the presence of O_2_ and NADPH, these enzymes oxidize the fatty acid molecule and produce NADP and H_2_O. However, this system is unable to introduce double bonds beyond position 9 (ω9). For this reason, fatty acids with double bonds in positions 3 and 6 (linolenic acid and linoleic acid) must be supplied through the diet and are known as essential fatty acids. The parallel metabolic synthesis of these essential fatty acid families, along with the distinct downstream lipid mediators and cellular mechanisms through which ω-3 PUFAs modulate inflammatory and lipogenic pathways, is schematically integrated in Figure 1.

The complexity of these molecular pathways underscores that the clinical efficacy of ω-3 PUFAs is strictly dependent on their effective incorporation into target tissues. Therefore, a comprehensive understanding of their biological mechanisms necessitates the use of standardized biomarkers, such as the Omega-3 Index, to bridge the gap between theoretical molecular potency and actual clinical outcomes.

## 3. Biomarkers and Indices for Ω-3 Measurement

### 3.1. Biological Matrices and Kinetic Profiles

The determination of ω-3s could theoretically be performed on any biological sample, though the choice of matrix significantly influences the clinical interpretation. Plasma concentrations of ω-3s primarily reflect recent dietary or supplemental intake. Within the plasma lipid fraction, ω-3s are incorporated into lipoproteins, meaning their concentrations fluctuate based on post-absorptive kinetics: chylomicrons last about 1 h, VLDL 4–6 h, and LDL/HDL 3–5 days. ω-3s in erythrocyte membranes persist for about 120 days [51].

To bypass the lengthy and complex nature of standard plasma or erythrocyte fatty acid analysis, alternative methods have been proposed. Among these, the nitrogen isotope ratio (^15^N/^14^N expressed as δ^15^N), which remains stable in nature, has been suggested as a surrogate marker of EPA and DHA intake. δ^15^N is high in Inuit populations. Erythrocyte δ^15^N correlates well with erythrocyte EPA and moderately with DHA, though DHA shows a plateau effect. Differences may arise because erythrocyte EPA and DHA reflect intake over the past two weeks, while nitrogen turnover reflects about 120 days. EPA is distributed on the outer membrane layer with faster turnover, whereas DHA is in the inner layer [52]. Beyond individual fatty acid tracking, another approach involves determining ω-6 and ω-3 scores and their ratios. A low AA:EPA ratio has been proposed as a desirable biomarker profile for cardiovascular risk reduction, and a low EPA/AA ratio has been used as an enrichment criterion in interventional trials (see RESPECT-EPA, Section 6) [53].

### 3.2. Clinical Significance and Cardiovascular Risk Protection

A low intake of EPA and DHA is a well-established driver of cardiovascular mortality. In randomized controlled trials in secondary prevention, fish and fish oil have been shown to reduce cardiovascular mortality at a dose of about 1 g per day [54]. The clinical utility of measuring these fatty acids is best exemplified by the ω-3 Index—defined as the proportion of EPA and DHA in erythrocytes—which functions as an independent cardiovascular risk factor, especially for sudden cardiac death [55]. An ω-3 Index ≥8% appears cardioprotective, while <4% is associated with increased cardiovascular risk [5]. Since its original proposal in 2004 as a risk factor for sudden cardiac death, the ω-3 Index has undergone progressive validation [56]. An updated global map (2024), based on 342,864 participants from 48 countries, confirmed that the large majority of the world’s population has low or deficient ω-3 Index levels, with 75% of countries lacking representative data [57]. In elite athletes, the mean ω-3 Index was 4.43%, with less than 1% of subjects in the optimal range (8–11%) [58]. A recent review emphasizes that an ω-3 Index in the 8–11% range is associated with lower total mortality and fewer major adverse cardiovascular events [59]. In light of this evidence, the earlier characterization of the ω-3 Index as “promising, though not yet fully validated” does not reflect the current state of the literature and is revised accordingly. It should nonetheless be noted that, although no clinical guideline currently recommends routine measurement of the ω-3 Index in clinical practice, the growing body of evidence supports its utility as a risk biomarker and as a potential tool to optimize the response to supplementation in the setting of clinical research [59,60].

The clinical predictive power of this biomarker is highlighted by the fact that the hazard ratio for coronary heart disease decreases by 15% per 1-SD increase in ω-3 Index [61]. Sudden cardiac death (SCD) is one of the most relevant endpoints in evaluating ω-3 PUFAs as cardioprotective agents. The ω-3 Index was proposed as a risk factor for SCD as early as 2004 [5], and a systematic meta-analysis of observational studies documented that higher EPA + DHA erythrocyte-membrane or higher plasma phospholipid levels of EPA + DHA + DPA are associated with a 45% reduction in SCD risk (pooled HR 0.548) [62]. Mechanistically, EPA and DHA are thought to stabilize cardiomyocyte membranes by modifying ion-channel properties—particularly the late sodium and calcium currents—reducing vulnerability to malignant ventricular arrhythmias. In the REDUCE-IT trial, no significant effect on SCD emerged as a separate endpoint, but the reduction in total cardiovascular mortality is consistent with an antiarrhythmic effect of EPA [63]. Similar inverse correlations between baseline ω-3 Index and mortality were observed in the Framingham Offspring Study [64].

These biomarker trends align with large-scale epidemiological data. In people with diabetes or prediabetes from the UK Biobank, fish oil supplementation was associated with a 9% and 13% lower rate of cardiovascular events, respectively [65].

Furthermore, data from the GAPP study documented that ω-3 intake correlates with the ω-3 Index and inversely with the albumin/creatinine ratio, suggesting cardio-renal protection [66]. ω-3 intake also lowers triglycerides and non-HDL cholesterol [61,67].

Ultimately, across studies, considerable uncertainty remains about the preventive role of ω-3s in cardiovascular disease, partly due to the lack of baseline EPA and DHA measurements in many trials.

## 4. Ω-3 Formulations and Absorption

One source of uncertainty in defining the cardioprotective effects of ω-3s comes from differences in how they are consumed: foods, fortified foods, or supplements. Supplements may contain various mixtures of polyunsaturated fatty acids (PUFAs) and can be provided as triglycerides or ethyl esters. Formulation affects bioavailability and the ability to achieve an ω-3 Index associated with risk reduction.

Bioavailability is the extent to which an active ingredient is absorbed from a medicine and becomes available in the body after being released, digested and absorbed.

For ω-3s, it is important to distinguish between acute bioavailability, meaning the concentration of the traced product measured in the blood after administration, and chronic bioavailability, meaning the amount of ω-3s detectable in tissues following administration [36]. Conventionally, this measure is obtained using red blood cell membranes (a tissue easily collected with a blood draw) [68]. This distinction is important because the pharmacological effects of EPA and DHA are not acute or immediately dependent on intake, but rather result from the incorporation and accumulation of these substances into cell membranes.

In food, ω-3s are consumed as triglycerides, which are digested in the intestine by lipases. Many commercial products are in ethyl ester form (a type not naturally occurring). PUFA absorption is better from triglycerides than from ethyl esters, so re-esterified triglyceride formulations are preferred. Modern re-esterification techniques can produce PUFA-rich triglycerides, especially high in DHA [69]. Labels identify ethyl esters with “EE” and triglycerides with “TG” or “rTG”.

Currently, ALA is the only ω-3 with a Dietary Reference Intake (DRI), although DHA and EPA are incorporated more efficiently into erythrocytes [70], ALA and SDA are only partly converted to EPA and do not provide DHA; EPA can serve as a precursor for DHA [71], although earlier literature suggests otherwise [61]. Women convert ALA to EPA/DHA more efficiently than men and may show a greater ω-3 Index response with age and post-menopause [72]. Once absorbed, ω-3s circulate as triglycerides (EPA and DHA) and phospholipids (EPA).

Beyond the fatty acid profile, the oxidative stability of the formulation is a key determinant of both biological efficacy and gastrointestinal tolerability. Because of their highly unsaturated structure, long-chain ω-3 PUFAs are intrinsically prone to lipid peroxidation, and susceptibility increases with the number of double bonds, so that DHA (six double bonds) oxidizes more readily than EPA (five) [73]. Oxidation proceeds in two phases. Primary oxidation generates lipid hydroperoxides, quantified by the peroxide value (PV); these unstable intermediates subsequently decompose during secondary oxidation into aldehydes, ketones, and other carbonyl compounds, captured by the *p*-anisidine value (*p*-AV). The total oxidation value (TOTOX = 2 × PV + *p*-AV) integrates both phases and is the most widely used composite index of oil freshness. The rate and extent of oxidation are governed by exposure to oxygen, light, and elevated temperature, and by storage time, whereas added antioxidants (e.g., tocopherols) slow the process [73]. Consumption of oxidized oil is undesirable on several grounds: secondary peroxidation products such as malondialdehyde (MDA) may blunt the pro-resolving signalling of EPA- and DHA-derived mediators, lower the effective delivered dose of intact PUFAs, and worsen organoleptic quality and gastrointestinal tolerability.

Oxidative quality in the marketplace is frequently suboptimal: in a survey of 171 over-the-counter ω-3 products from 49 North American brands, 50% exceeded at least one voluntary oxidation limit, with 39% above the TOTOX limit, 41% above the *p*-AV limit, and 17% above the PV limit [74]. No single, universally mandated cut-off exists; instead, the principal industry bodies—including the Global Organization for EPA and DHA ω-3 PUFAs (GOED), the Council for Responsible Nutrition, and the International Fish Oil Standards (IFOS) programme—converge on the same voluntary maximal limits of PV ≤ 5 mEq/kg, *p*-AV ≤ 20, and TOTOX ≤ 26 [73,74], against which independent third-party programmes such as IFOS certify batch-level compliance [75]. These specifications, together with trans-isomer considerations, are addressed in detail in the *Quality, Oxidation and Trans-Isomer Considerations* Section.

Natural sources of ω-3 such as fish provide triglyceride or phospholipid forms. Plant sources provide triglycerides as well. After refinement, ω-3s may be re-esterified with ethanol (ethyl esters, common in pharmaceuticals) or glycerol (triglycerides, sometimes concentrated). Algae provide mostly DHA. Other sources include herring roe, cod liver oil, and mussels, which contain more DHA than EPA [76,77]. Genetically modified plants rich in EPA/DHA are under study but not yet used in human nutrition [68]. Seeds provide ALA, but its conversion to EPA is limited and varies by sex.

ω-3s are available as capsules, emulsions, and microencapsulated forms. Capsules are most common, usually made of fish or bovine gelatine, and may be designed to release content in the alkaline intestinal environment. Microencapsulation allows controlled release and food fortification, increasing the surface available to lipases.

After mechanical dispersion by peristalsis, lipid particles—triglycerides, phospholipids, ethyl esters, or waxes—are digested in the small intestine by specific enzymes (ethyl ester digestion is 10–50 times slower than triglycerides and requires bile salts and a high-fat meal) [39]. Once absorbed by enterocytes, fatty acids are re-esterified and packaged into a lipoprotein called a chylomicron to be secreted in the lymphatic system. A critical and frequently underestimated aspect is the marked inter-individual variability in ω-3 bioavailability, which depends largely on the subject’s baseline ω-3 status—reflected by the erythrocyte ω-3 Index or plasma EPA + DHA levels—and on dietary habits, particularly oily fish consumption [36]. Subjects with a low baseline ω-3 status show a more pronounced response to supplementation than those with already elevated levels; this variability may partly explain differences in efficacy across populations with different dietary habits, as in the REDUCE-IT/JELIS comparison [63,78]. A further factor is the fat content of the accompanying meal: co-administration with a high-fat meal stimulates biliary and pancreatic secretion, enhancing the lipolysis of ethyl esters—whose hydrolysis by pancreatic lipase is 10–50 times slower than that of triglycerides [36]. Acute bioavailability studies have shown that intake of ethyl esters with a high-fat meal (44 g of fat) significantly increases plasma EPA levels compared with a low-fat meal (8 g) [36]. It is therefore advisable that ethyl-ester-based supplements be taken with meals.

### Quality, Oxidation and Trans-Isomer Considerations

**Figure 2:**
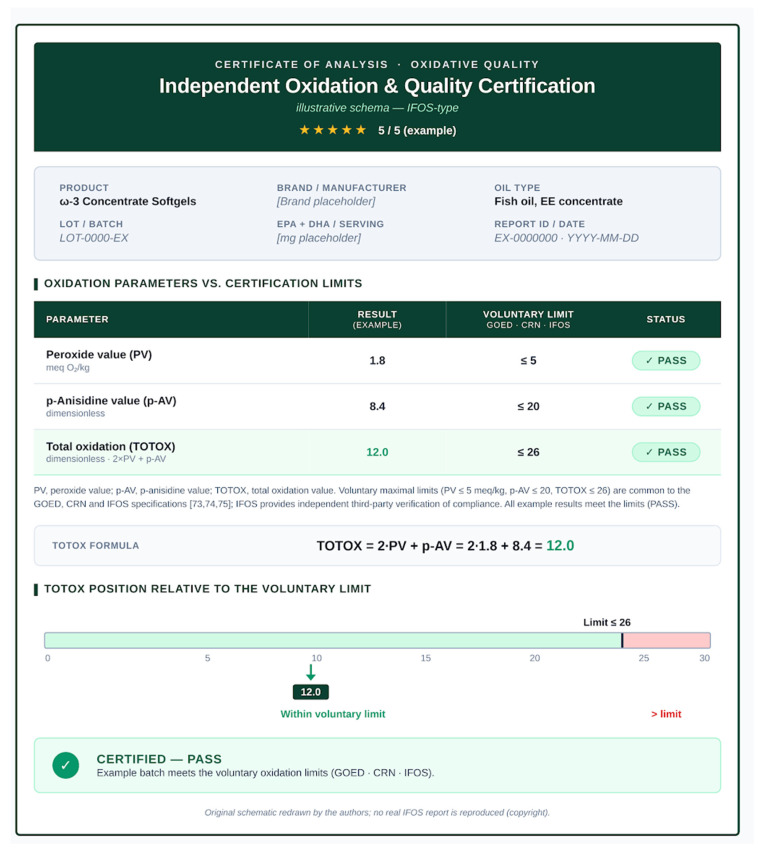
Illustrative schema of an independent oxidative-stability certification report (IFOS-type) for an ω-3 product. To pass, a product must simultaneously meet the voluntary oxidation limits endorsed across the sector: PV ≤ 5 mEq/kg, *p*-AV ≤ 20, and TOTOX ≤ 26 (TOTOX = 2 × PV + *p*-AV). These limits are common to the GOED, CRN and IFOS specifications; Original schematic redrawn by the authors; no real report is reproduced.

The clinical efficacy and tolerability of ω-3 products depend not only on dose and chemical form but also on manufacturing quality, an aspect not captured by efficacy trials. To address oxidative quality, independent certification programs have emerged, with the IFOS being the most widely recognized [75]. To earn IFOS certification, a product must simultaneously meet the sector’s voluntary oxidation limits: a peroxide value (PV) ≤ 5 mEq/kg, a *p*-anisidine value (*p*-AV) ≤ 20, and a total oxidation value (TOTOX) ≤ 26. These criteria align perfectly with the specifications of the GOED [73] (Figure 2). The added value of independent third-party certification lies not in stricter numerical thresholds but in batch-level verification of compliance, which directly addresses the market-level non-compliance discussed in the previous section; the underlying oxidative chemistry is detailed there.

A further quality consideration, not yet addressed by current certification schemes, is the geometric isomerization of ω-3 PUFAs. The native *cis* double bonds of EPA and DHA can partially convert to the *trans* configuration during refining, where steam distillation at high temperature (160–200 °C) is used to remove odorous aldehydes, ketones, and free fatty acids [79]. Because conventional steam deodorization is applied to the oil in triglyceride form, upstream of any transesterification to ethyl esters, it represents one of the critical steps for *trans*-isomer formation during manufacturing. Ferreri et al. were the first to chemically synthesize EPA mono-*trans* isomers as analytical reference standards and to detect them in deodorized fish oil; in a rat model, these isomers were incorporated into hepatic mitochondrial membranes following dietary intake, although their relevance to human tissues and outcomes remains unknown to date [80]. Comparing several refining/deodorization techniques, Song et al. reported significant differences in the resulting *cis*/*trans* profile (*p* < 0.05), indicating that the choice of process directly influences the degree of isomerization [81].

From a regulatory standpoint, the WHO recommends limiting total *trans* fatty acid intake to less than 1% of total daily energy (<2.2 g/day in a 2000 kcal diet) [82]. It should be emphasized that the reported increases in all-cause (+34%) and coronary heart disease (+28%) mortality associated with high *trans*-fat intake derive from epidemiological data on total dietary (largely industrial) *trans* fats [83] and are not transferable to the mono-*trans* LC-PUFA isomers discussed here, for which no human outcome data currently exist. Importantly, pharmaceutical-grade preparations (e.g., IPE) and independently certified supplements are manufactured under controlled, pharmacopoeia-compliant processes and contain negligible amounts of *trans* isomers, well below the WHO threshold. The presence of *trans* isomers should therefore be regarded as a manufacturing-quality issue and an argument in favour of independent certification that merits further targeted quantitative study, rather than a demonstrated clinical risk of ω-3 therapy.

An example of the oxidation profile and quality certification of an ω-3 sample is shown in Figure 2.

## 5. From Marine to Algal Sources: Nutritional Quality, Bioavailability, and Environmental Sustainability

The massive adoption of intensive breeding in recent times has led to a significant reduction in the ω-3 content in many products including field crops, eggs and meat [84]. Over the last 30 years, advancements in plant genetic engineering have introduced biofortified foods; specifically, researchers have successfully ‘reverse-engineered’ crops to synthesize long-chain ω-3 PUFAs, creating products suitable for both human and animal consumption [85].

Despite these innovations, fish remains the primary source of ω-3 PUFAs in human nutrition. However, the final EPA and DHA content and their subsequent bioavailability are significantly influenced by culinary practices, fishing methods, and aquaculture conditions. Thermal processing, particularly pan-frying, can lead to the oxidative degradation of polyunsaturated fats. Leung et al. demonstrated that frying increases lipid peroxidation markers, such as malondialdehyde (MDA), compared to boiling or baking [86]. In addition, the choice of cooking oil is also relevant; frying in oils rich in ω-6 fatty acids, such as sunflower oil, can exacerbate the loss of EPA and DHA due to lipid exchange and oxidation processes [87]. Recent data indicate that the ω-3 to ω-6 ratio can decrease threefold during deep-frying compared to raw fish, primarily due to the high absorption of frying oil, although the frying temperature itself appears to have a negligible impact on this ratio [88]. Overall, from the perspective of lipid nutritional quality, baking and steaming are the most recommended cooking methods for fish. Notably, even short-term refrigeration (exceeding 6 days) has been shown to adversely affect EPA + DHA concentrations [87].

Aquaculture and fishing methods also play a decisive role in the nutritional profile of the end product. As early as 1990, Van Vliet et al. reported that farmed fish generally contained lower ω-3 levels than wild-caught counterparts [89]. More recently, Sprague et al. observed a significant decline in the EPA and DHA content of farmed fish between 2006 and 2015 [90]. This trend is closely tied to shifting aquaculture practices: while feeds rich in marine-derived ingredients (such as microalgae and phytoplankton) support high tissue ω-3 levels, economic pressures have prompted a transition toward plant-based feeds. The resulting inclusion of ω-6-rich vegetable oils in fish diets has led to a progressive dilution of the ω-3 content in farmed species [91].

The increasing demand for ω-3 intake, through both fish consumption and fish oil supplementation, necessitates a critical evaluation of its global environmental impact. Intensive fish farming poses significant ecological risks, including waste-related pollution, the use of medications and fertilizers, and the potential for nutrient overload leading to harmful algal blooms [91,92]. Aquaponic systems have emerged as a potential alternative; by combining aquaculture with hydroponics in a symbiotic relationship, fish waste serves as fertilizer for plants, which in turn purify the water. This closed-loop system significantly mitigates the environmental footprint of traditional aquaculture, which is often characterized by high water and land consumption and substantial greenhouse gas emissions [93]. Nevertheless, widespread implementation of aquaponics faces several challenges, including high initial investment and operational costs, intensive energy requirements, and the need for specialized technical expertise. Furthermore, potential system failures and limitations in the selection of suitable fish and plant species currently restrict its scalability as a primary source for global ω-3 production [94].

Modern commercial fishing, even when employing more sustainable techniques such as hook-and-line, remains an inadequate solution. It contributes to significant plankton depletion and exerts a detrimental impact on marine ecosystems [95]. Furthermore, the global reliance on large-scale trawlers has led to severe overfishing, while the unintentional capture of non-target species—known as bycatch—further threatens biodiversity [96].

Consequently, there is a growing advocacy for plant-based sources of ω-3. While oilseed products (such as flaxseed, hemp, and sunflower oil) are rich in α-linolenic acid (ALA), their clinical utility is limited by the slow and inefficient conversion of ALA into EPA and DHA. In contrast, microalgae oils and plant-based seafood analogs have emerged as highly sustainable and relevant sources of long-chain ω-3s [97,98,99,100].

Microalgae (such as *Phaeodactylum tricornutum*, *Laminaria japonica* and *Crypthecodinium cohnii* are unicellular organisms widely distributed in marine and freshwater environments. As the primary producers in the marine food web, they are the original source of EPA and DHA, capable of efficient *de novo* synthesis with faster growth rates than terrestrial plants or animals [101]. Microalgae can be cultivated in controlled, land-independent systems that require minimal freshwater and generate significantly lower greenhouse gas emissions; specifically, algal oil production yields up to 50% fewer CO_2_ emissions per unit of ω-3 compared to both wild-caught and farmed fish oil [102]. Furthermore, microalgae cultivation eliminates risks associated with bycatch, ocean habitat degradation, and the accumulation of environmental contaminants [103].

Some studies indicate that algae-derived oil is both environmentally more sustainable and safer. Unlike marine-derived oils, which are subject to the biomagnification of heavy metals and persistent organic pollutants, algal oils are produced in pristine, controlled environments [72,73,74]. While some pharmaceutical companies employ advanced purification to remove contaminants from fish oil, emerging concerns regarding microplastics (MPs) further favor algal sources. Although evidence is still evolving, fish-derived oils—especially those from smaller fish—may harbor higher concentrations of MPs [104,105,106,107,108].

Market analyses also highlight a growing consumer preference for vegan supplements, with the global market projected to reach nearly USD 3 billion by 2030, driven by concerns over ocean health and ethical, cruelty-free dietary choices [109].

In parallel, food technology innovation has enabled the rise in plant-based seafood alternatives, which replicate the organoleptic and nutritional properties of traditional seafood using seaweed, legumes, and algal oils as key ingredients. Once again, these products offer a favorable sustainability profile, reducing greenhouse gas emissions by up to 87% and drastically lowering land and freshwater usage compared to conventional seafood [110].

Innovations in 3D printing and fermentation are further expanding this frontier, allowing for the creation of novel textures and nutrient profiles without the need for animal sacrifice [111]. From a nutritional perspective, algal-derived EPA and DHA are bioequivalent to fish oil and often retain essential vitamins, minerals, and antioxidants.

In conclusion, plant-based ω-3s and seafood alternatives represent a scalable, ethical, and evidence-based solution to global nutritional needs. Continued investment in production optimization and public education will be essential to enhance the accessibility and acceptance of these sustainable innovations.

A comprehensive overview of the current landscape of ω-3 PUFA options is provided in Table 3, which presents a comparative matrix of available ω-3 PUFA sources and formulations. Specifically, the table outlines their biochemical forms, key characteristics, advantages, and disadvantages, while cross-referencing intestinal bioavailability with critical environmental sustainability parameters such as carbon footprint, overfishing, bycatch, and contaminant risks [112,113,114].

**Table 3:** Comparative matrix of available ω-3 PUFA sources and formulations. The table explicitly details the biochemical form, key characteristics, advantages, and disadvantages of each option, cross-referencing their intestinal bioavailability with environmental sustainability parameters (carbon footprint, overfishing, bycatch, and contaminant risks).

Source/Formulation	Biochemical Form	Key Characteristics	Advantages (Bioavailability & Nutrition)	Disadvantages & Risks	Environmental & Sustainability Impact
Wild-Caught Fish *(e.g., herring, cod)*	Natural Triglycerides (TG) and Phospholipids (PL)	Intact food matrix; delivers preformed EPA and DHA.	High acute and chronic bioavailability. Provides co-nutrients (proteins, Vitamin D, Selenium).	Dietary restrictions (not vegan/vegetarian) Culinary degradation (frying lowers ω-3\ω-6 ratio) [86].	High ecological footprint due to overfishing and trawling [96]. Contributes to bycatch and biodiversity loss [95].
Farmed Fish *(Aquaculture)*	Natural Triglycerides (TG)	Controlled breeding; historical primary source of commercial fish oil.	Good baseline bioavailability. Stable and predictable market supply.	Progressive ω-3 dilution over the last decades due to plant-based feeds [89]. Higher ω-6 content [91].	High risk of waste pollution, medication runoff, and harmful algal blooms [91]. Aquaponics mitigates this but faces scalability limits [94].
Plant Sources *(e.g., flaxseed, chia)*	Triglycerides containing ALA	Rich in Alpha-Linolenic Acid (ALA; C18:3 ω-3).	Strictly vegan/vegetarian friendly. Excellent baseline oxidative stability.	Extremely poor conversion of ALA to EPA and DHA [97].	Highly sustainable; significantly lower carbon footprint compared to marine animal sources [97].
Algal Oil Supplements	Triglycerides (TG)	Concentrated preformed PUFAs directly synthesized by microalgae.	Mostly provides DHA. Bioequivalent to fish oil in terms of absorption. Vegan/vegetarian alternative.	Often more expensive than standard fish oils. Historically limited availability of high-EPA forms.	Highly sustainable; yields up to 50% fewer CO2 emissions vs. fish oil [102]. Land-independent and free from microplastics, heavy metals and bycatch risk [103].
Plant-Based Seafood Analogs	Matrix fortified with Algal Oils	Replicates traditional seafood using legumes, seaweed, and algal oils.	Replicates organoleptic properties without animal sacrifice. Often enhanced by 3D printing or fermentation.	Highly processed food matrix. Variable nutritional profiles depending on the brand.	Exceptional sustainability profile; reduces greenhouse gases by up to 87% vs. traditional seafood [110].
Ethyl Esters (EE)	Synthetic Esters	Synthetic form; fatty acids trans-esterified to an ethanol backbone.	Allows highly concentrated, standardized pharmaceutical doses. Cost-effective production.	10–50 times slower digestion by pancreatic lipase [39]. Strictly dependent on a high-fat meal.	Requires multi-step industrial manufacturing, adding chemical synthesis steps to the fish oil supply chain.
Re-esterified Triglycerides (rTG)	Re-esterified TGs	Industrial conversion of Ethyl Esters back into a glycerol backbone.	Superior bioavailability compared to EE. Does not require a high-fat meal for optimal absorption.	Complex and more expensive manufacturing process. High peroxidation risk (TOTOX) if not properly stabilized.	High carbon and processing footprint due to the intensive multi-step chemical concentration process.

## 6. Cardiovascular Evidence of ω-3 PUFAs

Epidemiological studies that highlighted an association between the consumption of ω-3 PUFAs from fish in the diet and a lower rate of cardiovascular disease and mortality in the past led to great expectations from the use of ω-3s for the reduction in cardiovascular risk.

In reality, many trials and meta-analyses in the following years failed to demonstrate significant cardiovascular benefits with the use of mixed formulations of EPA and DHA [115].

On the contrary, the JELIS trial on a Japanese population had demonstrated a statistically significant reduction in major coronary events with the administration of 1800 mg EPA plus statin compared to statin administration alone, in patients with a history of coronary heart disease [78].

Based on these data, the REDUCE-IT trial was set up. It was a randomized, double-blind, placebo-controlled trial, involving patients with established cardiovascular disease or with diabetes and other risk factors, who had been receiving statin therapy and who had a fasting triglyceride level of 135 to 499 mg per deciliter (1.52 to 5.63 mmol per liter) and a low-density lipoprotein cholesterol level of 41 to 100 mg per deciliter (1.06 to 2.59 mmol per liter). The patients were randomly assigned to receive 2 g of icosapent ethyl twice daily (total daily dose, 4 g) or placebo. The primary end point was a composite of cardiovascular death, nonfatal myocardial infarction, nonfatal stroke, coronary revascularization, or unstable angina. EPA as IPE (icosapent ethyl) at 4 g/day reduced ASCVD events by 25% compared with placebo (HR 0.75; 95% CI 0.68–0.83; *p* < 0.001) [63].

The results of this trial, whose findings were in contrast with those of other RCTs with EPA + DHA, have been much discussed, also in light of the possibility that the mineral oil comparator in REDUCE-IT had an adverse effect on cardiovascular risk, rather than EPA having a beneficial effect. Effectively, the administration of mineral oil was associated with increases in LDL-C (7.4%), apolipoprotein B (6.7%) and high-sensitivity C-reactive protein (37.6%), at last visit, relative to IPE. Nevertheless, the REDUCE IT data were independently reviewed by the FDA, Health Canada, the European Medicines Agency and the Committee for Medicinal Products for Human Use. On the basis of a review of the literature on mineral oil and post hoc analyses of data from REDUCE-IT, it was concluded that the increase in levels of the biomarkers with mineral oil would explain only a small percentage of the 25% difference in MACEs between the two randomised groups [116].

However, a re-evaluation was subsequently carried out of serum levels of interleukin-1β, interleukin-6, high-sensitivity C-reactive protein, oxidized low-density lipoprotein cholesterol, homocysteine, lipoprotein(a), and lipoprotein-associated phospholipase A2 (Lp-PLA2) at baseline, at 12 months, at 24 months, and at the end-of-study visit among REDUCE-IT participants. The new data confirmed that the levels of biomarkers associated with atherosclerosis increased over time among those allocated to mineral oil treatment. The effect of these findings on the interpretation of the REDUCE-IT trial results remains unclear [117].

This debate is further complicated by the results of the STRENGTH trial, which investigated a carboxylic acid formulation of EPA and DHA using corn oil as a neutral placebo [118]. Unlike REDUCE-IT, the STRENGTH study was terminated early for futility, as it failed to demonstrate any significant reduction in major adverse cardiovascular events (MACE). The discrepancy between these two large-scale trials underscores the ongoing uncertainty regarding whether the diverging outcomes are attributable to the different chemical formulations (purified EPA vs. EPA + DHA), the achieved plasma EPA levels, or the potential non-neutral effects of the mineral oil comparator used in REDUCE-IT.

After the REDUCE-IT study, also the EVAPORATE study (Effect of Vascepa on Progression of Coronary Atherosclerosis in Persons With Elevated Triglycerides, 200–499 mg/dL, on Statin Therapy) with IPE 4 g/day, as an adjunct to diet and statin therapy, demonstrated a significant regression of low-attenuation plaque (LAP) volume measured by serial multidetector computed tomography (MDCT), by 17%, while in the placebo group LAP volume more than doubled (+109%) (*p* = 0.0061) [119].

A further and more recent prospective, multicenter, randomized, open-label, blinded end-point study, RESPECT-EPA, evaluated patients with stable coronary artery disease and a low EPA/AA ratio (<0.4), who were randomized to EPA (1800 of IPE administered daily) or a control group [53].

The primary end point was a composite of cardiovascular death, nonfatal myocardial infarction, nonfatal ischemic stroke, unstable angina pectoris, and coronary revascularization.

The addition of a low EPA/AA ratio (<0.4) as an inclusion criterion was based on reports indicating destabilization of coronary artery plaques and an increase in coronary events in patients with CAD with an EPA/AA < 0.4. Patients in the control group were not administered placebo, as its absence could eliminate the concern of any prognostic effect of placebo, after the intense debate on mineral oil as a placebo in the REDUCE-IT trial.

The median baseline plasma EPA level in this cohort was 48 µg/mL, lower than that observed in the JELIS study (97 µg/mL) [78] but notably higher than in the REDUCE-IT study (26 µg/mL). This underscores the persistent disparity in dietary fish intake between Japanese and Western populations.

As a consequence, the median plasma EPA concentration in Western individuals taking 4000 mg of IPE in REDUCE-IT (144 µg/mL) was comparable to that in Japanese individuals taking 1800 mg of IPE in the RESPECT-EPA study (140 µg/mL), even if it did not reach the level of the JELIS study (169 µg/mL) [53]. The heterogeneity of results across the large cardiovascular ω-3 trials cannot be fully understood without considering two often-overlooked methodological variables: the baseline plasma levels of EPA and DHA in the enrolled population, and the pharmacokinetic complexity of these endogenous nutrients [120]. Unlike xenobiotic drugs, EPA and DHA are already present in the body in variable amounts before pharmacological intervention; randomization to treatment does not guarantee that subjects reach therapeutic tissue levels, and a neutral trial result may reflect insufficient incorporation into target tissues rather than a true lack of efficacy [59]. This concept is exemplified by the REDUCE-IT/JELIS comparison discussed above, in which comparable plasma EPA concentrations were reached at very different administered doses owing to differences in baseline ω-3 status. A systematic analysis suggests that an ω-3 Index in the optimal range (8–11%) is associated with lower total mortality and reduced major cardiovascular events, and that the failure to stratify by baseline ω-3 status may contribute to the heterogeneity of trial results [59]. It should be emphasized that this interpretive framework, although supported by robust observational data, is not yet incorporated into any clinical guideline as an indication for routine ω-3 Index measurement in current clinical practice.

Over a median period of 5 years, IPE treatment resulted in a numerically lower risk of cardiovascular events but without reaching statistical significance for the primary endpoint. Conversely, the incidence of the secondary composite end point of coronary events, which included sudden cardiac death, myocardial infarction, unstable angina, and coronary revascularization, was significantly lower in the EPA group than in the control group.

Furthermore, an additional per-protocol analysis that excluded patients with drug discontinuation or protocol violation showed that IPE treatment was significantly associated with reduced risk of the primary end point and secondary composite end point of coronary events. The authors acknowledged that their study was probably underpowered because of the low overall rate of cardiovascular events compared to the REDUCE-IT population.

To provide an overview of current research, the table below (Table 4) highlights the relationship between commonly used ω-3 PUFA dosages and their observed clinical outcomes.

## 7. Official Indications of ω-3 PUFAs

ω-3 PUFAs currently have limited but well-defined therapeutic indications. The strongest evidence concerns the use of IPE in reducing residual cardiovascular risk in high-risk patients treated with statins and with moderately elevated triglycerides, as demonstrated by the REDUCE-IT study and incorporated into major international guidelines. Another indication is the treatment of severe hypertriglyceridemia for the prevention of pancreatitis.

In recent years, several studies have suggested that the potential clinical applications of ω-3 PUFAs may extend beyond reducing hypertriglyceridemia and the cardiovascular area. Indeed, their anti-inflammatory and immunomodulatory properties have shown promising preliminary results in psychiatric, neurological, autoimmune, and other conditions [123,124,125,126].

Despite the growing interest in a wider use, ω-3 PUFAs have only a limited number of indications nowadays. These approved uses are confined primarily to cardiovascular prevention in selected patient populations and to the management of severe hypertriglyceridemia.

### 7.1. Cardiovascular Risk Reduction in High-Risk Patients with Elevated Triglycerides

The most significant therapeutic indication for ω-3 PUFAs derives from the results of the REDUCE-IT trial [63], which provided evidence that high-dose purified EPA, as IPE, reduces residual cardiovascular risk in statin-treated patients with elevated triglycerides.

In December 2019, the U.S. Food and Drug Administration (FDA) approved IPE as an adjunct to maximally tolerated statin therapy to reduce the risk of cardiovascular events in adults with established atherosclerotic cardiovascular disease (ASCVD) or with diabetes mellitus and at least one other cardiovascular risk factor, provided fasting triglyceride levels were ≥150 mg/dL [127]. This represented the first approval of an ω-3 formulation for cardiovascular event reduction, beyond its lipid-lowering effect. The EMA followed in 2021, authorizing IPE for adults with established ASCVD who are receiving statins and have fasting triglycerides ≥150 mg/dL [128].

Scientific societies incorporated these findings into practice guidelines. The 2019 ESC/EAS Guidelines for the Management of Dyslipidaemias recommend considering IPE in high- and very-high-risk patients with triglycerides between 135–499 mg/dL who remain above target despite statin therapy [129], and the recent Focused Update confirmed this recommendation [130]. Similarly, the 2021 ACC Expert Consensus Decision Pathway on the Management of Hypertriglyceridemia emphasizes that purified EPA is the only ω-3 product with proven benefit in reducing ASCVD risk when added to statins [131].

The American Diabetes Association (ADA) Standards of Care 2026 recommend that in individuals with ASCVD or other cardiovascular risk factors on a statin with managed LDL-C but elevated triglycerides (150–499 mg/dL), the addition of IPE can be considered to reduce cardiovascular risk [132]. While previous documents, such as the 2017 AHA Science Advisory, considered EPA + DHA treatment reasonable for secondary prevention [133], the 2023 AHA/ACC Guidelines for the Management of Chronic Coronary Disease have significantly updated this stance [134]. These current guidelines explicitly advise against the use of over-the-counter fish oil and ω-3 supplements, classifying them as having no proven benefit for cardiovascular event reduction (Class 3). Regarding heart failure, the 2022 AHA/ACC/HFSA Heart Failure guidelines recognize ω-3 PUFAs as a therapeutic option, with a Class 2b recommendation, in patients with symptomatic heart failure (NYHA II–IV) to reduce mortality and cardiovascular hospitalization [135]. In contrast, the European guidelines maintain a more conservative stance: both the 2021 ESC Guidelines for the diagnosis and treatment of acute and chronic heart failure [136] and their 2023 Focused Update [137] do not recommend ω-3 PUFAs for this indication.

These recommendations establish high-purity EPA as the standard of care for triglyceride-associated residual risk reduction in high-risk patients on statins. ω-3 formulations containing DHA have not demonstrated cardiovascular benefits in large-scale outcome trials and are therefore excluded from these indications.

### 7.2. Severe Hypertriglyceridemia and Pancreatitis Prevention

The other recognized indication for ω-3 PUFAs is the management of severe hypertriglyceridemia (≥500 mg/dL). Both EPA + DHA and pure EPA formulations are approved for this indication.

The FDA first approved ω-3 acid ethyl esters in 2004 for the treatment of severe hypertriglyceridemia to reduce the risk of pancreatitis. The EMA has approved several prescription ω-3 formulations for the same indication, typically at doses of 2–4 g/day.

The 2019 ESC/EAS Guidelines on Dyslipidaemias recommended ω-3 (2–4 g/day of EPA + DHA) as an adjunct to diet in patients with severe hypertriglyceridemia, particularly when triglyceride levels exceed 500 mg/dL, to lower the risk of acute pancreatitis [129].

## 8. Safety and Adverse Reactions

From a safety standpoint, ω-3s are generally well tolerated; however, pharmacological doses have been associated with an increased risk of atrial fibrillation and, to a lesser extent, bleeding, with a favorable risk-benefit profile in appropriately selected patients.

### 8.1. Atrial Fibrillation

One of the most consistent safety signals associated with high-dose ω-3 therapy is the increased risk of atrial fibrillation (AF). Several RCTs and a meta-analysis of intervention trials have highlighted a dose-dependent increased risk of atrial fibrillation in patients with known cardiovascular disease or multiple cardiovascular risk factors treated with high doses of ω-3 (up to 4 g/day) [2,138].

In the REDUCE-IT trial, the incidence of AF was significantly higher in the IPE group (5.3%) compared with placebo (3.9%) [63], a finding mirrored in the STRENGTH trial, where 2.2% of patients receiving ω-3 presented AF, compared with 1.3% in the placebo group [118].

The OMEMI trial, which included elderly post-MI patients, observed a non-significant trend toward increased AF incidence in those receiving ω-3 supplementation [121].

Collectively, a meta-analysis of 7 large RCTs, including more than 80,000 participants, confirmed that ω-3 supplementation is associated with a 25% increased risk of AF, regardless of the formulation used, with a progressive increase of approximately 11% for each additional gram of ω-3 administered daily [138].

These findings led the EMA in September 2023 to recommend an update of the information material, advising the permanent suspension of treatment in case of the onset of AF [139].

In contrast, the VITAL-Rhythm trial, which tested the long-term effects of marine ω-3 PUFAs (approx. 460 mg/d of EPA and 380 mg/d of DHA) in a large-scale RCT of individuals without prior vascular disease, found no significant difference in the risk of incident AF over a median follow-up of more than 5 years [122].

This is consistent with broader epidemiological data; for instance, a meta-analysis of 17 prospective cohort studies involving 55,000 subjects found that higher blood or adipose tissue levels of ω-3 (EPA, DPA, and DHA) were not associated with increased AF risk. Conversely, a reduced risk was observed in subjects with higher levels of DPA and DHA [140]. Similarly, an analysis of UK Biobank data in over 261,000 subjects found that plasma ω-3 levels were inversely associated with AF incidence [141].

This apparent discrepancy may be explained by the substantial difference between nutritional and pharmacological dosages and by the baseline risk profile of the populations. Most cohort participants were community-dwelling individuals at relatively low CV risk, whose biomarker levels reflected habitual dietary intake rather than high-dose supplementation.

Furthermore, the timeline to observe potential benefits may be longer than the duration of typical RCTs. Habitual ω-3 intake exerts long-term influence on multiple upstream risk factors, such as blood pressure, type 2 diabetes, systemic inflammation, and chronic sympathetic activation, potentially offering a protective effect that cannot be feasibly captured in shorter intervention trials [140].

From a mechanistic standpoint, the relationship between ω-3 PUFAs and AF appears to follow a non-linear, potentially biphasic pattern. The effects of ω-3s on the sodium and calcium channels of cardiomyocytes can lead to a shortening of the action potential duration and a slowing of conduction, which may trigger both antiarrhythmic and pro-arrhythmic effects depending on the concentration. It has been suggested that at low doses, vagally mediated effects reduce AF risk through membrane stabilization. However, at progressively higher pharmacological doses, heightened vagal tone can induce sinus bradycardia and increase the risk of AF [142].

Although the absolute risk increase is modest, AF remains clinically relevant due to the association with systemic embolism and stroke. Given that risk factors for AF, such as increasing age, hypertension and obesity, are very common in patients with hypertriglyceridemia [143], some clinicians highlight the need for a careful prescription of ω-3 in susceptible individuals [144].

**Figure 3:**
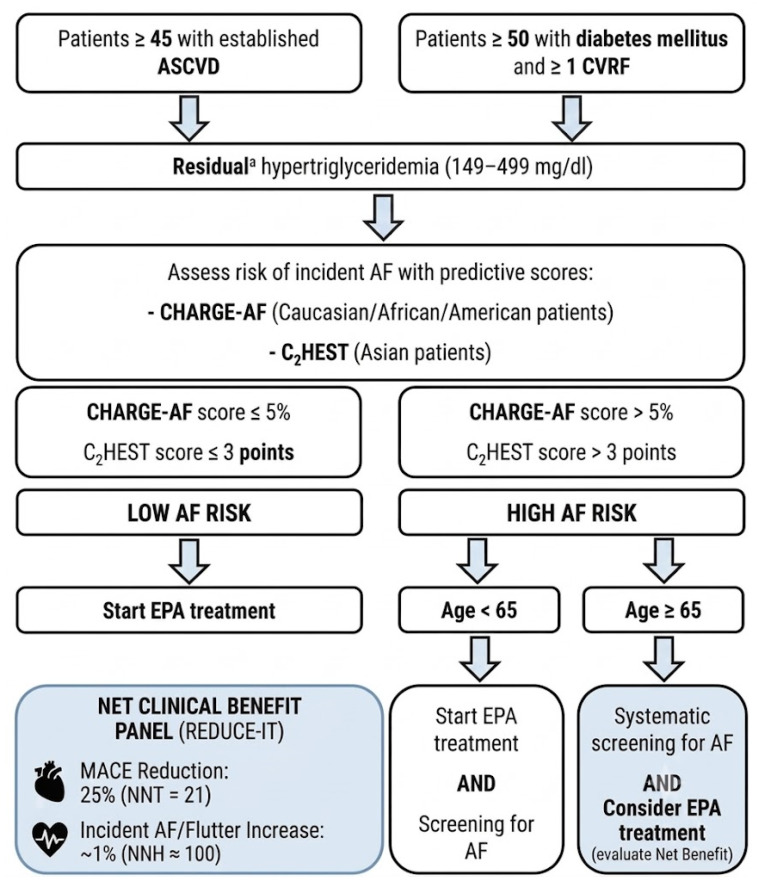
Suggested flowchart to guide EPA prescription on the basis of AF risk. ^a^ Residual hypertriglyceridemia despite optimal or maximally tolerated statin therapy. The net clinical benefit panel incorporates REDUCE-IT trial data, highlighting the favorable balance between MACE reduction (NNT = 21) and incident AF/flutter increase (NNH ≈ 100). ASCVD: atherosclerotic cardiovascular disease; CVRF: cardiovascular risk factor; EPA: Eicosapentaenoic acid, 4 g/day (high-dose regimen); AF: Atrial Fibrillation; MACE: Major Adverse Cardiovascular Events; NNT: Number Needed to Treat; NNH: Number Needed to Harm.

Subgroup analyses suggest that patients older than 65 may derive lower net benefit from high-dose EPA due to their higher baseline AF risk [63]. Consequently, these susceptible individuals should undergo systematic screening for atrial arrhythmias, whereas low-risk subjects may not require such intensive monitoring. To guide clinicians, a flowchart for the safe management of EPA therapy has been proposed (Figure 3), facilitating the identification of candidates for high-dose regimens based on their specific AF risk profile [2,145]. The AF risk associated with ω-3 fatty acids is dose-dependent and cannot be assessed in isolation from the overall benefit profile. An updated meta-analysis of 34 randomized trials (114,326 participants) found that a statistically significant increase in AF risk was confined to patients at high cardiovascular risk treated with high doses (>1500 mg/day of EPA and/or DHA), in whom the pooled odds ratio was 1.48 (95% CI, 1.21–1.81; absolute risk difference ≈ 0.8%); no significant increase was observed at doses achievable through diet or standard supplements (≤1500 mg/day), irrespective of baseline risk [146]. Notably, when all trials were pooled together, AF risk remained modestly but significantly elevated (≈14%), indicating that the null finding is restricted to the lower-dose and lower-risk strata rather than reflecting the absence of any class effect [146]. A dose-dependent, vagally mediated mechanism has been proposed to reconcile these divergent effects: at the low doses attainable through diet, ω-3 fatty acids increase vagal tone and lower resting heart rate without raising—and possibly lowering—AF risk, whereas at high pharmacological doses the same autonomic and electrophysiological changes may become pro-arrhythmic, yielding a “double-edged sword” intake–risk relationship [142,146]. This is consistent with the U-shaped relationship between ω-3 intake and AF depicted by Fatkin et al. [142,147]. Crucially, the risk–benefit balance must incorporate hard clinical and mortality endpoints. In REDUCE-IT, the ≈1.0-percentage-point absolute increase in hospitalization for atrial fibrillation/flutter (3.1% vs. 2.1%) was offset by a 4.8-percentage-point absolute reduction in the primary composite endpoint (25% relative reduction; hazard ratio 0.75) and by a 20% relative reduction in cardiovascular death (hazard ratio 0.80; 4.3% vs. 5.2%) [63]. Expressed as numbers needed to treat, one primary endpoint event was prevented for every 21 patients treated over a median 4.9 years (95% CI, 15–33) [63]; conversely, based on the reciprocal of the observed absolute AF excess, approximately 100 patients would need to be treated for one additional AF hospitalization. The number needed to harm for AF is therefore substantially higher—i.e., more favorable—than the number needed to treat for a major cardiovascular event, supporting a clear net clinical benefit in appropriately selected high-risk patients [148].

### 8.2. Bleeding Risk

Given their antiplatelet effects, ω-3 PUFAs have raised concerns regarding bleeding complications. In REDUCE-IT, serious bleeding events occurred in 2.7% of patients in the IPE group compared with 2.1% on placebo, a difference close to statistical significance (*p* = 0.06) [63].

The STRENGTH trial did not report an excess of major bleeding [118]. A meta-analysis of 11 RCTs, including more than 120,000 patients, concluded that ω-3 supplementation did not significantly increase the risk of any bleeding events. When data were restricted to trials with IPE, high-dose purified EPA was associated with a 50% higher risk of any bleeding compared with control, but a modest absolute increase in risk (0.6%) [149].

In clinical practice, ω-3s are not contraindicated in patients taking antiplatelet or anticoagulant therapy; clinicians should exercise caution, particularly when prescribing high-dose, concentrated EPA/DHA formulations to individuals at high risk for hemorrhage, regardless of whether they are derived from marine sources or microalgal bioprocessing. Rather than relying solely on a generic recommendation for caution, some authors suggest that the bleeding risk could be further quantified and contextualized. From a pathophysiological standpoint, an increased bleeding susceptibility manifests mainly at very high ω-3 Index levels and appears related to the effect of EPA on the inhibition of platelet aggregation [59]. Consequently, a clinically useful approach proposed for patients at high hemorrhagic risk who are candidates for high-dose therapies is the measurement of the ω-3 Index. This may assist in monitoring systemic exposure and identifying subjects with very high erythrocyte EPA + DHA levels who might carry an incremental bleeding risk.

Crucially, this potential bleeding risk is dictated by the specific fatty acid profile of the source rather than its animal or botanical origin, establishing a clear distinction between long-chain PUFAs (EPA/DHA) and short-chain PUFAs (ALA) [150]. Clinically relevant antiplatelet and antithrombotic effects, mediated by the displacement of arachidonic acid in platelet membranes and the subsequent reduction in thromboxane A2 synthesis, are exclusive to high-dose EPA and DHA [150]. Consequently, this biological effect applies equally to both marine-derived fish oils and plant-based microalgal oils, as microalgae naturally synthesize large amounts of preformed, highly bioavailable DHA and EPA [151].

Conversely, terrestrial plant oils (such as flaxseed, chia, or hemp oil) rich in ALA do not increase bleeding susceptibility or alter primary hemostasis. This safety profile is directly attributable to the highly inefficient and limited conversion cascade of ALA into EPA/DHA in humans, which fails to reach the high systemic and intra-platelet thresholds required to modulate platelet aggregation [152].

### 8.3. Other Adverse Effects

The most common adverse effects of ω-3 supplementation are gastrointestinal and include dyspepsia, diarrhea, nausea, and fishy aftertaste. These side effects are generally mild, transient, dose-dependent, and rarely lead to treatment discontinuation [153]. Enteric-coated formulations and administration with meals can mitigate these symptoms.

Formulations containing EPA and DHA have consistently been associated with modest increases in LDL-C (typically 5–10%), particularly at doses ≥3 g/day [25,154]. This paradoxical effect may offset some of the benefits of triglyceride reduction, especially in patients with mixed dyslipidemia. Mechanistic studies suggest that DHA is the primary driver of LDL-C elevation, possibly through enhanced conversion of VLDL to LDL and alterations in hepatic LDL receptor activity [155]. By contrast, EPA-only formulations have shown a neutral effect on LDL-C [63,156]. This quantitative increase in LDL-C does not necessarily reflect a greater atherogenic risk. Studies of LDL subpopulations have shown that ω-3 PUFAs—particularly EPA + DHA combined with statins—produce a favorable shift from the small, dense LDL fraction (sdLDL), highly atherogenic owing to its susceptibility to oxidation and arterial-wall retention, toward large, buoyant LDL particles (lbLDL), which are intrinsically less atherogenic [157]. The net result is an increase in total LDL-C concentration alongside a reduction in the overall pro-atherogenic activity of the LDL particles. This distinction is clinically relevant because it clarifies why the LDL-C increase observed with DHA + EPA is not associated with a proportional increase in cardiovascular risk.

ω-3 PUFAs supplementation correlates with a modest increase in HDL, generally 3–5% [158], and to a shift in HDL subfractions, with enrichment of large HDL particles, which are considered more cardioprotective [159].

In summary, ω-3 PUFAs are well tolerated. Pharmacological doses are associated with a small but consistent increase in AF, and a modest trend toward increased bleeding. These risks, however, are outweighed by the demonstrated cardiovascular benefits in appropriately selected high-risk patients.

**Figure 4:**
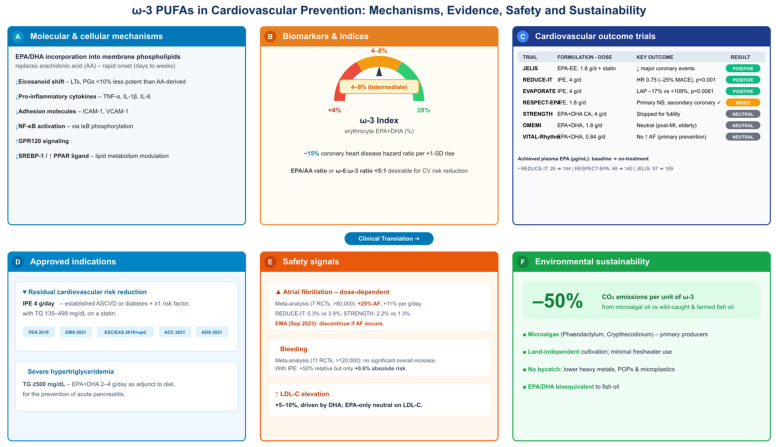
Graphical summary of the main benefits and results of ω-3 PUFAs in cardiovascular prevention: molecular and cellular mechanisms; biomarkers; cardiovascular outcome trials with achieved plasma EPA; approved indications; dose-dependent safety signals and the sustainability advantage of microalgal oils. Upward arrows (↑) indicate an increase or up-regulation; downward arrows (↓) indicate a decrease, reduction, or risk reduction; horizontal arrows (→) denote clinical translation or progression. Abbreviations: AA, arachidonic acid; AF, atrial fibrillation; ASCVD, atherosclerotic cardiovascular disease; DHA, docosahexaenoic acid; EE, ethyl ester; EPA, eicosapentaenoic acid; HR, hazard ratio; IPE, icosapent ethyl; LAP, low-attenuation plaque; LDL-C, low-density-lipoprotein cholesterol; MACE, major adverse cardiovascular events; POPs, persistent organic pollutants; SD, standard deviation; TG, triglycerides.

The principal mechanisms, biomarker thresholds, cardiovascular outcome trials, approved indications, safety signals, and sustainability considerations discussed throughout this review are summarized in Figure 4.

## 9. Conclusions

ω-3 polyunsaturated fatty acids represent a nutritionally and pharmacologically relevant class of bioactive lipids, with well-characterized molecular mechanisms spanning anti-inflammatory signaling, membrane remodeling, eicosanoid metabolism, and transcriptional regulation of lipid homeostasis. From a clinical standpoint, current evidence supports specific, well-defined therapeutic indications: high-dose purified EPA (as IPE) significantly reduces residual cardiovascular risk in statin-treated patients with elevated triglycerides, as established by the REDUCE-IT trial and reflected in international guidelines. While these formulations remain a cornerstone for managing severe hypertriglyceridemia, safety data from large randomized trials indicate a modest but consistent increase in atrial fibrillation risk at pharmacological doses. This arrhythmic potential warrants individualized risk stratification and underscores the necessity of a ‘biomarker-guided’ approach to supplementation.

Rather than relying on standardized oral dosing, the therapeutic target should be defined by objective metrics—such as plasma EPA and DHA concentrations, the ω-3 Index, or the EPA/AA ratio. Such a tailored strategy effectively accounts for the significant heterogeneity driven by geographical provenance, habitual fish consumption, and global culinary practices, which ultimately dictate fatty acid bioavailability and integrity. Furthermore, the transition from ‘mixed’ ω-3 formulations to high-purity EPA represents a paradigm shift in cardiovascular prevention, requiring future research to prioritize the standardization of bioavailability protocols that account for chemical forms (TG vs. EE) and dietary context.

Finally, beyond clinical efficacy, the growing environmental burden of marine-derived production calls for a critical reassessment of sourcing strategies. Microalgae-based oils and plant-based seafood alternatives offer promising sustainable options, with baseline nutritional profiles comparable to fish-derived products and significantly lower ecological footprints. However, given the established clinical superiority of purified EPA over DHA-containing mixtures for cardiovascular risk reduction, future biotechnological efforts must specifically focus on developing and scaling high-EPA microalgal strains. Bridging the gap between the pharmacological demand for EPA and the historically DHA-dominant profile of microalgae will be crucial. Continued investment in research, production optimization, and regulatory frameworks will be essential to translate these biotechnological innovations into scalable public health solutions, balancing potent anti-atherosclerotic benefits against clinical safety and global environmental responsibility.

## Figures and Tables

**Table 1:** Contribution of ω-3 PUFAs from representative dietary sources. Content varies markedly with species, wild vs. farmed origin, season, and culinary preparation; approximate representative values are listed.

Food	ALA (g/100 g)	EPA + DHA (g/100 g)	EPA + DHA per Typical Portion (mg)	% of 250 mg/day AI	Ref.
Salmon, Atlantic (cooked)	–	~2.0	~3000 (150 g)	~1200%	[14]
Mackerel, salted (cooked)	–	~4.6	~6900 (150 g)	~2740%	[1]
Herring, Atlantic (cooked)	–	~2.0	~3000 (150 g)	~1200%	[14]
Sardines (canned, drained)	–	~1.0	~1500 (150 g)	~600%	[14]
Tuna, light (canned in water)	–	~0.2	~300 (150 g)	~120%	[14]
Cod, Atlantic (cooked, lean fish)	–	~0.16	~240 (150 g)	~95%	[14]
Cod liver oil	–	~19	~850 (1 tsp, ~4.5 g)	~340%	[14]
Flaxseed (linseed) oil	45–55	nil	nil	—	[13]
Flaxseed (whole/ground)	20–24	nil	nil	—	[14]
Chia seeds	16–19	nil	nil	—	[14]
Walnuts (English)	8–10	nil	nil	—	[14]
Canola oil	8–10	nil	nil	—	[14]
Soybean oil	6–8	nil	nil	—	[14]

**Table 2:** ω-3 (EPA + DHA) intake recommendations across scientific and health organizations. Abbreviations: AHA, American Heart Association; ANSES, Agence nationale de sécurité sanitaire de l’alimentation, de l’environnement et du travail; CHD, coronary heart disease; DHA, docosahexaenoic acid; EFSA, European Food Safety Authority; EPA, eicosapentaenoic acid; FAO, Food and Agriculture Organization; ISSFAL, International Society for the Study of Fatty Acids and Lipids; SACN, Scientific Advisory Committee on Nutrition; VKM, Norwegian Scientific Committee for Food Safety; WHO, World Health Organization.

Organization	Population	Recommended EPA + DHA	Notes	Ref.
EFSA (2010)	General adults	250 mg/day	Adequate intake; +100–200 mg/day DHA in pregnancy/lactation; 100 mg/day DHA for infants 6 mo–2 y	[18]
WHO/FAO (2010)	General adults	≥250 mg/day	Minimum for cardiovascular health; basis for global intake gap analyses	[24]
ISSFAL (Third Statement)	Adults, cardiovascular health	≥500 mg/day	Minimum intake for maintenance of cardiovascular health	[19]
ANSES (France)	Adults	500 mg/day	National reference intake	[20]
SACN (UK, 2004)	Adults	≥450 mg/day	≈2 portions of fish/week, of which ≥1 oily	[21]
AHA (Rimm et al., Science Advisory 2018)	Secondary CHD prevention	~1 g/day	≥2 fish servings/week for the general population	[25]
Health Council of the Netherlands	Adults	~200 mg/day	Lower end of national recommendations	[17]

**Table 4:** Most commonly used doses of ω-3 PUFAs in clinical trials and clinical outcomes.

Study/Context (Year)	Formulation	Dose	Population/Indication	Outcome	Ref.
JELIS (2007)	Highly purified EPA ethyl ester (ethyl icosapentate, ~96–98% EPA) ^a^	1800 mg/day + statin	Hypercholesterolemia (primary and secondary prevention, predominantly primary); Japanese population	↓ Major coronary events (statistically significant)	[78]
REDUCE-IT (2019)	IPE (highly purified EPA ethyl ester)	4000 mg/day (2 g × 2)	ASCVD or diabetes + ≥1 risk factor; fasting TG 135–499 mg/dL	↓ 25% MACE (HR 0.75, 95% CI 0.68–0.83; *p* < 0.001) ^b^	[63]
EVAPORATE (2020)	IPE	4000 mg/day	Statin-treated; coronary plaque; TG 200–499 mg/dL	LAP regression −17% vs. +109% placebo (*p* = 0.0061)	[119]
RESPECT-EPA (2023)	IPE	1800 mg/day	Stable CAD; EPA/AA ratio <0.4	Primary endpoint NS; secondary coronary composite significantly ↓	[53]
STRENGTH (2020)	Carboxylic acid EPA + DHA	4000 mg/day	High cardiovascular risk	Terminated early for futility (neutral; no MACE reduction)	[118]
OMEMI (2021)	EPA + DHA	1.8 g/day total (930 mg EPA + 660 mg DHA) ^c^	Elderly post-MI patients	Neutral (non-significant trend toward ↑ AF)	[121]
VITAL-Rhythm (2021)	EPA + DHA (marine)	460 mg EPA + 380 mg DHA/day (840 mg/day)	Primary prevention; no prior vascular disease	No increase in incident AF (neutral)	[122]

^a^ JELIS used ethyl icosapentate, a highly purified EPA ethyl ester (~96–98% EPA), i.e., the same molecular entity as icosapent ethyl (IPE). ^b^ REDUCE-IT primary endpoint: composite of cardiovascular death, nonfatal myocardial infarction, nonfatal stroke, coronary revascularization, or unstable angina. Guideline/regulatory entries are reported for context and do not represent trial efficacy endpoints (n/a). ^c^ OMEMI total ω-3 dose of 1.8 g/day corresponds to 930 mg EPA + 660 mg DHA (1590 mg EPA + DHA). Symbols: ↓ reduction or decrease in clinical events/endpoints; ↑ increase or upward trend. Abbreviations: AA, arachidonic acid; AF, atrial fibrillation; ASCVD, atherosclerotic cardiovascular disease; CAD, coronary artery disease; CI, confidence interval; DHA, docosahexaenoic acid; EPA, eicosapentaenoic acid; HR, hazard ratio; IPE, icosapent ethyl; LAP, low-attenuation plaque; MACE, major adverse cardiovascular events; MI, myocardial infarction; NS, not significant; TG, triglycerides.

## Data Availability

No new data were created or analyzed in this study. Data sharing is not applicable to this article.
